# The association between douching, genital talc use, and the risk of prevalent and incident cervical cancer

**DOI:** 10.1038/s41598-021-94447-3

**Published:** 2021-07-21

**Authors:** Katie M. O’Brien, Clarice R. Weinberg, Aimee A. D’Aloisio, Kristen R. Moore, Dale P. Sandler

**Affiliations:** 1 Epidemiology Branch, National Institute of Environmental Health Sciences, 111 TW Alexander Dr., Research Triangle Park, NC 27709 USA; 2 Biostatistics and Computational Biology Branch, National Institute of Environmental Health Sciences, Research Triangle Park, NC USA; 3 Social and Scientific Systems, Inc., Durham, NC USA

**Keywords:** Cancer epidemiology, Risk factors, Cervical cancer

## Abstract

While human papillomavirus is the primary cause of cervical cancer, other factors may influence susceptibility and response to the virus. Candidates include douching and talcum powder applied in the genital area. We used Cox proportional hazards models to estimate confounder-adjusted hazard ratios (HRs) and 95% confidence intervals (CIs) in the Sister Study (2003–2009), a US cohort of women aged 35–74. We considered pre-baseline (n = 523) and incident (n = 31) cervical cancers. Douching at ages 10–13 was positively associated with pre-baseline cervical cancer (HR 1.32, 95% CI 0.86–2.03), though the association was not statistically significant. We did not observe an association between adolescent talc use and pre-baseline cervical cancer (HR 0.95, 95% CI 0.76–1.19). Douching in the year before enrollment was positively associated with incident cervical cancer (HR 2.56, 95% CI 1.10–5.99). The association between recent genital talc use and incident cervical cancer was positive, but not statistically significant (HR 1.79, 95% CI 0.78–4.11). The observed positive association between douching and incident cervical cancer is consistent with previous retrospective case–control studies. In the first study to examine genital talc use and cervical cancer, we did not see evidence of an association.

## Introduction

Cervical cancer is caused by human papillomavirus (HPV)^1^ and is largely preventable via inoculation^2–4^. Vaccines have been tied to substantial reductions in genital warts and other HPV-linked cervical abnormalities, though their impact on cervical cancer is not yet realized^5^. As of 2018, cervical cancer was the fourth most common cancer in women worldwide, representing 7% of all new cancers^6^.

HPV is a necessary but not sufficient cause of cervical cancer^7,8^, suggesting that other risk factors influence individuals’ susceptibility and response to the virus^9,10^. Identifying factors that determine whether cancerous lesions develop in HPV-exposed individuals may improve our understanding of the etiology of cervical cancer and other related conditions, and may lead to strategies for preventing cervical cancer in under-vaccinated regions.

Vaginal douching is one possible contributing factor. Women who douche may choose from a range of possible solutions, including water, homemade vinegar mixtures, or manufactured, chemically-scented products. Some of the solutions contain potentially carcinogenic chemicals, such as phthalates^11^ and volatile organic compounds^12^. Douching may also impact cancer risk by physically pushing pathogens up the reproductive tract^13^ or inhibiting immune response via alterations to the vaginal microbiome^14,15^.

In previous studies, douching has been positively associated with a range of outcomes, including bacterial vaginosis, increased susceptibility to sexually transmitted infections (STIs), infertility, and ectopic pregnancy^13,15–17^. Observational studies have documented associations between douching and HPV^18–20^, cervical lesions^21^, or progression of cervical lesions from low to high grade^22^. Additionally, several retrospective case–control studies have reported positive associations between douching and cervical cancer^23–28^. We did not identify any prospective studies of the relationship between douching and cervical cancer. Such studies are needed to rule out recall bias, which can result when individuals affected by a disease over-report their exposure to an agent of concern.

Genital talc use could also plausibly contribute to cervical cancer risk. Talc applied to underwear, sanitary napkins, diaphragms, or directly to the perineal region can enter the vagina and travel up the reproductive tract^29^. Talc particles may act as irritants, inciting an inflammatory response and potentially affecting individuals’ susceptibility and response to HPV infection^30^. Additional or more severe adverse effects could occur if the talc contained asbestos, a known carcinogen sometimes mined in the same locations as talc^31–33^. The epidemiologic literature supports a possible positive association between genital talc use and ovarian cancer^34,35^, and to a lesser extent uterine cancer^36–40^. To our knowledge, no studies have examined the relationship between genital talc use and cervical cancer. Here, we investigated the association of douching and genital talc use with cervical cancer in a large U.S.-based cohort.

## Methods

### Study sample

The Sister Study was designed to investigate the genetic and environmental causes of breast cancer and other chronic diseases in females from the United States^41^. Participants were 35–74 years of age at baseline (2003–2009), and had at least one sister with a history of breast cancer, but had never had breast cancer themselves.

Details on personal health, including any previous cancer diagnoses, were collected at baseline. We also collected data on demographic factors, health-related behaviors, and environmental and chemical exposures, including personal care product use. In-home examiners collected biologic samples and made anthropometric measures. Participants are being followed prospectively and are asked to report any major health changes, including cancer diagnoses, on annual follow-up surveys. More detailed health and exposure data are collected every 2–3 years, with response rates remaining around 90%.

The initial cohort included 50,884 women. We excluded women who were missing information on talc use and douching (n = 1365). For analysis of pre-baseline cervical cancer, we excluded women who withdrew (n = 3) or had uncertain cervical cancer diagnosis information (n = 8), leaving us with 49,508 women. An additional 523 women with pre-baseline cervical cancer were excluded from all analyses of incident cervical cancer (n = 48,985; data release 7.0; follow-up through 9/2017).

For the main analyses, we did not exclude person-time for women who had hysterectomies, as our questionnaires did not allow us to determine whether the cervix was also removed. However, we did conduct sensitivity analyses where we censored women at their age of hysterectomy. All participants provided written informed consent. The Sister Study was reviewed and approved by the institutional review boards of the National Institute of Environmental Health Sciences and the Copernicus group (IRB00001313).

### Exposure and outcome assessment

We asked participants to report douching and talc use at two time points: during early adolescence (ages 10–13) and in the 12 months prior to enrollment. At ages 10–13, participants reported whether they did not, sometimes, or frequently douched or used talc. For use in 12 months prior to enrollment, participants reported whether they douched or used talc: never, < 1 per month, 1–3 times per month, 1–5 times per week, or > 5 times per week. At both time points, genital talc use was specifically defined as the application of “talcum powder to a sanitary napkin, underwear, diaphragm, cervical cap, or directly to your vaginal area”. Though we include mostly time-specific analyses, we considered “ever” use to be use at ages 10–13 or in the year prior to enrollment.

We evaluated the risk of self-reported pre-baseline and incident cervical cancers separately. We did not systematically attempt to medically-confirm pre-baseline cases as they may have occurred many years prior to baseline, but incident cases were asked to provide medical documentation. Participants with medical documentation of a cervical cancer diagnosis or who had cervical cancer as a primary or underlying cause of death in National Death Index records were considered confirmed cases.

### Covariates of interest

We identified potential confounders using a direct acyclic graph framework^42^, selecting covariates that we hypothesized could be causally related to cervical cancer and talc or douching. When evaluating genital talc use or douching at ages 10–13, we considered weight relative to peers at age 10 (lighter, same weight, heavier), race/ethnicity (non-Hispanic White, non-Hispanic Black/African-American, Hispanic/Latina, other), childhood socioeconomic status (well off, middle income, low income, poor) and age at menarche (< 12, 12–13, > 13). In further-adjusted models, we additionally considered regular drinking before age 14 (yes/ no), smoking before age 14 (yes/no), and in utero diethylstilbestrol exposure (definitely/probably versus probably not/definitely not).

When estimating the potential effects of using douche or genital talc ever or in the year prior to enrollment, we adjusted for the following covariates, assessed at enrollment: race/ethnicity (non-Hispanic White/other), education (did/did not complete college), body mass index (BMI; continuous), and age at menarche (continuous). In further adjusted models we also considered marital status (ever/never married), age at first pregnancy (nulligravid, < 20, ≥ 20), ever induced abortion (yes/no), hormonal birth control use (< 2 years, ≥ 2 years), alcohol use (current/non-current drinker), smoking status (ever/never), ever diagnosed with genital warts, pelvic inflammatory disease (PID), or non-HPV STIs (gonorrhea, herpes, or chlamydia; yes/no). We did not have data on HPV infection or vaccination status, history of abnormal Pap smear or previous cervical treatments, or details about sexual history (e.g., age at sexual debut or number of sexual partners).

### Statistical analysis

We used multivariable Cox proportional hazards models to estimate hazard ratios (HRs) and 95% confidence intervals (CIs) for the association between history of douching or genital talc use and cervical cancer. Because the proportion of missing data was low (< 1%), we limited analyses to those with complete data for all covariates. We estimated variance using robust estimators to account for within-family clustering. We used the Breslow method^43^ to estimate the cumulative risk of cervical cancer and covariate-adjusted risk differences.

When assessing the association between douching or genital talc use and pre-baseline cervical cancer, we only considered use at ages 10–13 to ensure that exposure occurred prior to diagnosis. We started the time scale at age 13 and followed women until age at cervical cancer diagnosis, censoring at age at enrollment. Our use of retrospective data to reconstruct each woman’s pre-enrollment experience requires an assumption that a woman’s cervical cancer status did not influence her participation in the Sister Study.

We estimated the effects of ever use, frequency of use, and combined use. When examining frequency, we tested for trends by calculating p-values for the association between cervical cancer and an ordinal form of the exposure variable. For this and all other analyses, we tested the proportional hazards assumption by conducting a Wald test of the exposure-by-age interaction term.

We additionally examined whether the associations between pre-baseline cervical cancer and adolescent douching or genital talc use may be modified by certain covariates, including the following: race/ethnicity (non-Hispanic White versus other), follow-up time (≤ 20 years versus > 20 years), self-reported average BMI ages 30–39 (< 25 kg/m^2^ versus ≥ 25 kg/m^2^), history of any STI (yes versus no previous diagnosis of genital warts, gonorrhea, herpes, chlamydia, or PID), age at first pregnancy (nulligravid, age < 20, or age ≥ 20), and childhood socioeconomic status (well-off/middle income or low income/poor).

For our prospective analysis of genital talc use, douching and risk of incident cervical cancer, we started the age-time scale at age of study enrollment, censoring women at death, loss to follow-up, or end of follow-up. This analysis is statistically independent from the analysis of pre-baseline cervical cancer. We assessed douching and genital talc use ever and use in the year preceding enrollment, but did not examine adolescent use alone due to the rarity of this exposure and the small number of incident cases. Similarly, we did not consider analyses restricted to medically-confirmed incident cases or frequency of use.

To examine the potential impact of cervical cancer status misclassification, we conducted bias analyses where we selected a random sample of either 40% or 20% of the unconfirmed cases to be re-coded as non-events. All pre-baseline cases were considered unconfirmed. We conducted such analyses for the following associations: (1) douching ages 10–13 and pre-baseline cervical cancer; (2) genital talc use ages 10–13 and pre-baseline cervical cancer; (3) douching in the year before enrollment and incident cervical cancer; and (4) genital talc use in the year before enrollment and incident cervical cancer. This re-sampling and re-analysis process was repeated 20 times for each association of interest, then summarized using imputation software (PROC MIANALYZE; SAS 9.4). All methods were performed in accordance with the relevant guidelines and regulations.

### Ethics approval and consent to participate

This analysis made use of data collected previously as part of the Sister Study and is covered by the ethics review conducted by the Institutional Review Board of the National Institute of Environmental Health Sciences, National Institutes of Health and the Copernicus Group Institutional Review Board (IRB00001313). Data access was approved by Dr. Dale Sandler, Principal Investigator of the Sister Study. All participants provided written informed consent.

### Consent for publication

All participants signed an informed consent term for data publication.

## Results

After excluding participants with missing covariates, our main analysis included 49,302 women, 523 of whom reported a cervical cancer diagnosis prior to enrollment. Forty-one women self-reported incident cervical cancer. Twenty-six of those provided medical documentation, of which 16 were confirmed (positive predictive value = 62%). Fifteen others who did not provide documentation were assumed to be true cases, including one confirmed via the National Death Index. As such, there were 31 incident cervical cancer cases, including 17 medically-confirmed. The 10 falsely-reported cases were re-assigned to be non-cases; 6 had other cancers (5 endometrial/uterine, 1 in situ vulva and vaginal) and 4 had non-cancerous cervical conditions (3 dysplasia, 1 high-grade squamous intraepithelial lesion).

Compared to non-cases, women with pre-baseline cervical cancer tended to be less educated (Table 1; 39% with at least a college degree, versus 51% of non-cases), more likely to have had an abortion (21% versus 15%), more likely to be ever smokers (65% versus 44%), and more likely to have been diagnosed with genital warts, an STI, or PID (16%, 17%, 7%, respectively, in pre-baseline cases, versus 6%, 12% and 4% in non-cases).

**Table 1:** Covariate distributions according to cervical cancer diagnosis status among 49,508 women participating in the Sister Study cohort (2003–2009) [Excluded if: withdrawn from study (n = 3), cervical cancer status unknown (n = 3), age at diagnosis unknown (n = 4 cases), diagnosed before age 13 (n = 1) or did not fill out questionnaire on use of genital talc or douching products (n = 1365)].

	Non-cases (n = 48,954)	Pre-baseline cases (n = 523)	Incident cases (n = 31)
**Age at Baseline (years); mean (sd)**	55.7 (9.0)	55.6 (8.5)	54.0 (8.8)
**Baseline BMI (kg/m**^**2**^**); mean (sd)**	27.8 (6.2)	28.4 (7.2)	28.3 (6.7)
**BMI ages 30–39 (kg/m**^**2**^**); mean (sd)**	23.3 (4.0)	23.1 (3.7)	23.6 (4.3)
**Age at menarche (years); mean (sd)**	12.6 (1.5)	12.5 (1.6)	13.2 (1.2)
**Age at diagnosis (years); mean (sd)**		34.0 (9.2)	59.1 (9.1)
**Race; N (%)**			
Non-Hispanic White	41,177 (84)	447 (85)	26 (84)
Non-Hispanic Black/African-American	4140 (8)	30 (6)	5 (16)
Hispanic/Latina	2361 (5)	17 (3)	0 (0)
Other	1264 (3)	29 (6)	0 (0)
**Childhood socioeconomic status**^**a**^**; N (%)**			
Well off	3107 (6)	31 (6)	2 (6)
Middle income	29,175 (60)	288 (55)	20 (65)
Low income	12,682 (26)	151 (29)	6 (19)
Poor	3873 (8)	53 (10)	3 (10)
**Education; N (%)**			
High school or less	7542 (15)	91 (17)	5 (16)
Some college	16,464 (34)	226 (43)	11 (35)
College graduate	13,211 (27)	110 (21)	8 (26)
Graduate degree	11,728 (24)	96 (18)	7 (23)
**Marital status; N (%)**			
Never married	2624 (5)	31 (6)	3 (10)
Married/living as married	36,691 (75)	362 (69)	20 (65)
Divorced/widowed/separated	9628 (20)	130 (25)	9 (26)
**Pap smear in the year prior to enrollment; N (%)**	37,427 (77)	398 (76)	23 (74)
**Age at menarche; N (%)**			
< 12	9992 (20)	132 (25)	4 (13)
12–13	27,487 (56)	269 (51)	16 (52)
≥ 14	11,432 (23)	122 (23)	11 (35)
**Parity; N (%)**			
Nulliparous	8867 (18)	84 (16)	3 (10)
1 child	7047 (14)	94 (18)	2 (6)
2 children	18,011 (37)	185 (35)	14 (45)
> 2 children	14,999 (31)	159 (30)	12 (39)
**Age at first pregnancy; N (%)**			
Nulligravid	6145 (13)	54 (10)	1 (3)
< 20	9180 (19)	157 (30)	9 (29)
20–24	17,049 (35)	176 (34)	13 (42)
25–29	10,559 (22)	86 (16)	6 (19)
≥ 30	5932 (12)	49 (9)	2 (6)
**Induced abortion**	7533 (15)	107 (21)	10 (32)
**Duration of hormonal birth control use; N (%)**			
Never user	7431 (15)	68 (13)	2 (6)
0–< 2 years	7446 (15)	73 (14)	8 (26)
2–< 10 years	20,926 (43)	238 (46)	12 (39)
≥ 10 years	13,102 (27)	144 (28)	9 (29)
**Alcohol use; N (%)**			
Never or former drinker	9,320 (19)	90 (17)	4 (13)
Current drinker, < 1 drink/day	32,999 (67)	348 (67)	22 (71)
Current drinker, ≥ 1 drink/day	6616 (14)	85 (16)	5 (16)
**Drinking regularly ≤ age 13**	1988 (4)	32 (6)	5 (16)
**Smoking; N (%)**			
Never	27,575 (56)	182 (35)	10 (32)
Former	17,449 (36)	241 (46)	16 (52)
Current	3917 (8)	100 (19)	5 (16)
**Started smoking ≤ age 13**	1443 (3)	45 (9)	5 (16)
**Weight relative to peers, age 10; N (%)**			
Lighter	17,136 (35)	183 (35)	10 (32)
Same weight	22,914 (47)	226 (43)	13 (42)
Heavier	8873 (18)	114 (22)	8 (26)
**Ever diagnosed with genital warts; N (%)**	2863 (6)	82 (16)	2 (6)
Age at diagnosis; mean (sd)	28.9 (9.6)	29.4 (10.3)	42.0 (9.9)
**Ever diagnosed with an STI**^**b**^**; N (%)**	5651 (12)	88 (17)	5 (16)
Age at diagnosis; mean (sd)	29.2 (9.9)	29.5 (11.7)	29.2 (8.8)
**Ever had PID; N (%)**	1742 (4)	39 (7)	1 (3)
Age at diagnosis; mean (sd)	27.8 (8.0)	27.9 (8.6)	31.0 (0)
**Postmenopausal; N (%)**	32,588 (67)	352 (67)	20 (65)
**Hysterectomy; N (%)**	15,282 (31)	348 (67)	1 (3)
**Oophorectomy; N (%)**	8842 (18)	126 (24)	2 (6)
**Exposed to diethylstilbestrol in utero**^**c**^**; N (%)**	1115 (2)	32 (6)	0 (0)
**Family history of cervical cancer**^**d**^**; N (%)**	1854 (4)	47 (9)	3 (10)

*BMI* body mass index, *PID* pelvic inflammatory disease.

Missing values: BMI (15 non-cases), BMI ages 30–39 (399 non-cases, 2 pre-baseline cases, 1 incident case), age at menarche (43 non-cases), race/ethnicity (12 non-cases), childhood socioeconomic status (117 non-cases), education (9 non-cases), marital status (11 non-cases), Pap smear in the year prior to enrollment (37 non-cases, 2 pre-baseline cases), parity (30 non-cases, 1 pre-baseline case), age at first pregnancy (89 non-cases, 1 pre-baseline case), induced abortion (30 non-cases), duration of hormonal contraceptive use (49 non-cases), alcohol use (19 non-cases), started drinking alcohol regularly, age 13 (16 non-cases), smoking status (13 non-cases), started smoking ≤ age 13 (16 non-cases), weight relative to peers (31 non-cases), ever had genital warts (34 non-cases, 2 pre-baseline cases), ever had an STI (119 non-cases, 1 pre-baseline case), ever had PID (77 non-cases, 1 pre-baseline case), hysterectomy (23 non-cases), oophorectomy (75 non-cases, 2 pre-baseline cases).

^a^Defined as family’s income level during the majority of time growing up.

^b^STI = sexually transmitted infection, here defined as self-reported gonorrhea, herpes, chlamydia.

^c^If responded that mother “definitely” or “probably” took diethylstilbestrol; all others assumed to be unexposed.

^d^Had at least one first degree relative with cervical cancer (sister, mother, daughter).

The most striking differences between incident cases and either pre-baseline cases or non-cases were the proportion of Black/African-American women (16% of incident cases, 6% of pre-baseline cases, 8% of non-cases), nulligravid women (3% of incident cases, 10% of pre-baseline cases, 13% of non-cases) and women who had had an induced abortion (32% of incident cases, 21% of pre-baseline cases, 15% of non-cases).

Three percent of women reported douching in early adolescence, and 19% reported using genital talc at that time. In the year prior to enrollment, 14% douched and 15% used genital talc. Overall, 16% had ever douched and 28% had ever used genital talc.

Compared to those who neither douched nor used genital talc, women who douched were younger, had higher BMIs, were more likely to be Black/African-American, and were more likely to have had an induced abortion, smoked, or had an STI (Supplementary Tables 1 and 2). Women who douched or used genital talc had, on average, younger ages at menarche, compared to non-users. Genital talc users were more likely to be Black/African-American than non-users.

The estimated cumulative risk of developing cervical cancer by age 70 was 1.2%. For analyses of douching or genital talc use at ages 10–13 and pre-baseline cervical cancer, we included a mean of 42.5 years of follow-up (range 3.2–63.5). For incident cervical cancer, we followed women for a mean of 9.6 years (range 0.1–13.9).

In fully-adjusted models, we observed a positive but not statistically significant association between douching at ages 10–13 and pre-baseline cervical cancer (Table 2; HR 1.32, 95% CI 0.86–2.03), with a corresponding estimated adjusted risk difference of 0.36% (95% CI − 0.28% to 0.99%) at age 60. The effect estimates from the fully-adjusted models were attenuated compared to the more minimally-adjusted models, suggesting that there was some confounding by regular drinking before age 14, smoking before age 14, or in utero diethylstilbestrol exposure. When we censored women at age of hysterectomy, the effect estimates for douching were slightly stronger than those estimated in the main analysis, though still not statistically significant (e.g., ever versus never HR 1.49, 95% CI 0.97–2.29; Supplementary Table 3).

**Table 2:** Hazard ratios and 95% confidence intervals (CIs) for the associations between douching and genital talc use at ages 10–13 and cervical cancers diagnosed prior to enrollment [n = 49,302 (Participants with complete confounder information)].

	Person-time at risk (years)^a^	Non-cases; N(%) N = 48,779^b^	Cervical cancer cases, N(%) N = 523^b^	Age-adjusted hazard ratio (95% CI)	Adjusted hazard ratio^c^ (95% CI)	Fully adjusted hazard ratio^c,d^ (95% CI)
**Douching, ages 10–13**						
Ever use						
No	2,003,222	46,552 (97)	496 (96)	1.00	1.00	1.00
Yes	60,934	1525 (3)	22 (4)	1.40 (0.91, 2.14)	1.45 (0.94, 2.22)	1.32 (0.86, 2.03)
Frequency of use						
None	2,003,222	46,552 (97)	496 (96)	1.00	1.00	1.00
Sometimes	55,036	1378 (3)	19 (4)	1.34 (0.84, 2.11)	1.38 (0.87, 2.18)	1.26 (0.80, 2.00)
Frequently	5898	147 (0)	3 (1)	1.99 (0.64, 6.24)	2.16 (0.68, 6.82)	1.88 (0.59, 5.95)
				p-trend = 0.10	p-trend = 0.07	p-trend = 0.16
**Genital talc use, ages 10–13**						
Ever use						
No	1,580,656	36,905 (80)	399 (81)	1.00	1.00	1.00
Yes	393,069	9,131 (20)	96 (19)	0.97 (0.77, 1.21)	0.97 (0.77, 1.21)	0.95 (0.76, 1.19)
Frequency of use						
None	1,580,656	36,905 (80)	399 (81)	1.00	1.00	1.00
Sometimes	332,832	7,751 (17)	81 (16)	0.96 (0.76, 1.22)	0.96 (0.75, 1.22)	0.94 (0.74, 1.20)
Frequently	60,237	1,380 (3)	15 (3)	1.00 (0.59, 1.67)	1.00 (0.59, 1.69)	0.98 (0.58, 1.66)
				p-trend = 0.82	p-trend = 0.81	p-trend = 0.71
**Combined ever use, ages 10–13**						
Neither	1,543,967	36,006 (79)	389 (79)	1.00	1.00	1.00
Both	27,891	688 (2)	8 (2)	1.10 (0.55, 2.22)	1.15 (0.57, 2.31)	1.04 (0.52, 2.10)
Talc/no douching	356,534	8,238 (18)	87 (18)	0.97 (0.77, 1.23)	0.97 (0.76, 1.22)	0.95 (0.75, 1.21)
Douching/no talc	21,739	552 (1)	9 (2)	1.56 (0.81, 3.03)	1.62 (0.83, 3.16)	1.41 (0.72, 2.75)

Missing: douching (n = 702 non-cases, 5 cases), genital talc use (n = 2,743 non-cases, 28 cases), both (n = 3,295 non-cases, 30 cases).

^a^Participants with complete confounder information.

^b^With age as the primary time scale, adjusting for weight relative to peers at age 10 (ordinal), race/ethnicity (non-Hispanic White, non-Hispanic Black/African-American, Hispanic/Latina, or other), childhood socioeconomic status (well off, middle income, low income, poor), and age at menarche (< 12, 12–13 ≥ 14).

^c^Additionally adjusted for in utero diethylstilbestrol exposure (yes or no), regular drinking before age 14 (yes/no), and smoking before age 14 (yes/no).

The association between genital talc use and pre-baseline cervical cancer was null (Table 2; HR 0.95, 95% CI 0.76–1.19). Adjustment made minimal difference. Similarly, censoring women at their age of hysterectomy did not modify estimates (Supplementary Table 3). There was no evidence of a combined effect of douching and genital talc use on cervical cancer (HR 1.04, 95% CI 0.52–2.10).

We observed a violation of the proportional hazards assumption when assessing the relationship between adolescent douching and pre-baseline cervical cancer (p < 0.001). When we stratified by follow-up time, we observed a positive association during the first 20 years (Table 3; HR 1.75, 95% CI 1.07–2.87), but not after (HR 0.72, 95% CI 0.29–1.76, p-for-heterogeneity = 0.09). For the parallel assessment of adolescent genital talc use (p-for-proportional-hazards = 0.26), we saw a similar pattern (HR 1.19, 95% CI 0.89–1.59 for the first 20 years versus HR 0.70, 95% CI 0.48–1.01 for follow-up years ≥ 20, p-for-heterogeneity = 0.03). We did not observe heterogeneity in the estimated effects of douching or genital talc use by strata of race/ethnicity, BMI ages 30–39, STI status, age at first pregnancy, or childhood SES.

**Table 3:** Covariate-stratified hazard ratios and 95% confidence intervals (CIs) for the associations between douching and genital talc use at ages 10–13, and cervical cancer diagnosed prior to enrollment [n = 49,302 (Participants with complete confounder information)].

	Non-cases N = 48,779; N (%)	Cervical cancer cases N = 523^a^; N (%)	Douching adjusted hazard ratio^b^ (95% CI)	Genital talc use adjusted hazard ratio^b^ (95% CI)
**Race**				
Non-Hispanic White	41,069 (84)	447 (15)	1.32 (0.80, 2.19)	0.87 (0.67, 1.13)
Other	7,710 (16)	76 (85)	1.17 (0.51, 2.66)	1.23 (0.75, 2.02)
p-for-heterogeneity			0.81	0.22
**Follow-up time (starting age 13)**				
≤ 20 years	0 (0)	275 (53)	1.75 (1.07, 2.87)	1.19 (0.89, 1.59)
> 20 years	48,779 (100)	248 (47)	0.72 (0.29, 1.76)	0.70 (0.48, 1.01)
p-for-heterogeneity			0.09	0.03
**Body mass index at ages 30–39**				
< 30 kg/m^2^	37,981 (78)	420 (81)	1.25 (0.73, 2.12)	0.87 (0.66, 1.14)
≥ 30 kg/m^2^	10,420 (22)	101 (19)	1.55 (0.74, 3.26)	1.27 (0.82, 1.96)
p-for-heterogeneity			0.67	0.18
**STI status**^**c**^				
None	39,995 (82)	343 (66)	1.26 (0.72, 2.19)	0.86 (0.65, 1.14)
Any	8,780 (18)	180 (34)	1.27 (0.65, 2.51)	1.12 (0.77, 1.64)
p-for-heterogeneity			0.90	0.15
**Age at first pregnancy**				
Nulligravid	6,107 (13)	54 (10)	1.31 (0.29, 5.87)	1.16 (0.60, 2.27)
Gravid, < 20	9,138 (19)	157 (30)	1.04 (0.50, 2.17)	1.23 (0.84, 1.80)
Gravid, ≥ 20	33,448 (69)	311 (60)	1.50 (0.85, 2.65)	0.78 (0.57, 1.07)
p-for-heterogeneity			0.76	0.15
**Childhood SES**				
Well off or middle income	32,253 (66)	319 (61)	1.07 (0.58, 1.95)	0.98 (0.74, 1.31)
Low income or poor	16,526 (34)	204 (39)	1.73 (0.93, 3.22)	0.90 (0.61, 1.29)
p-for-heterogeneity			0.27	0.69

*STI* sexually transmitted infection, *SES* socioeconomic status.

Missing: douching (n = 702 non-cases, 5 cases), genital talc use (n = 2,743 non-cases, 28 cases), both (n = 3,295 non-cases, 30 cases), body mass index ages 30–39 (378 non-cases, 2 cases), STI status (4 non-cases), age at first pregnancy (86 non-cases, 1 case).

^a^Participants with complete confounder information.

^b^With age as the primary time scale, starting everyone at age 13 and censoring at age at baseline. Adjusted for weight relative to peers at age 10 (ordinal), age at menarche (< 12, 12–13, ≥ 14), race/ethnicity (non-Hispanic White, non-Hispanic Black/African-American, Hispanic/Latina, or other), childhood socioeconomic status (well off, middle income, low income, poor), regular drinking before age 14 (yes/no), smoking before age 14 (yes/no), and in utero diethylstilbestrol exposure (yes or no). If covariate is stratified on, it is not included in the model.

^c^Any sexually transmitted infection, including self-reported genital warts, gonorrhea, herpes, chlamydia, or pelvic inflammatory disease.

Ever douching (Table 4; HR 2.45, 95% CI 1.06–5.69) and douching in the year before enrollment (HR 2.56, 95% CI 1.10–5.99) were both positively associated with incident cervical cancer in fully-adjusted models. Again, the associations between douching and cervical cancer were stronger when we excluded post-hysterectomy person-time (Supplementary Table 4). The observed associations between genital talc use and cervical cancer were slightly positive, but imprecise and not statistically significant (HR 1.38, 95% CI 0.66–2.86 for ever use and HR 1.79, 95% CI 0.78–4.11 for use in year before enrollment).

**Table 4:** Hazard ratios and 95% confidence intervals (CIs) for associations between douching and genital talc use and incident cervical cancer (n = 48,509).

	Person-time (years)^a^	Non-cases; N(%) N = 48,478^b^	Incident cervical cancer cases, N(%) N = 31^b^	Age-adjusted hazard ratio (95% CI)	Adjusted hazard ratio^c^ (95% CI)	Fully-adjusted hazard ratio^c,d^ (95% CI)
**Ever douched**^**e**^						
No	378,771	40,495 (84)	20 (67)	1.00	1.00	1.00
Yes	70,421	7612 (16)	10 (33)	2.66 (1.25, 5.65)	2.64 (1.15, 6.09)	2.45 (1.06, 5.69)
**Douched in year prior to enrollment**						
No	389,309	41,547 (86)	21 (67)	1.00	1.00	1.00
Yes	59,628	6534 (14)	9 (33)	2.76 (1.28, 5.98)	2.72 (1.16, 6.35)	2.56 (1.10, 5.99)
**Ever genital talc use**^**e**^						
No	316,215	34,139 (72)	19 (66)	1.00	1.00	1.00
Yes	125,045	13,306 (28)	10 (34)	1.34 (0.62, 2.88)	1.39 (0.67, 2.89)	1.38 (0.66, 2.86)
**Genital talc use in year prior to enrollment**						
No	375,282	40,394 (85)	22 (76)	1.00	1.00	1.00
Yes	65,804	7031 (15)	7 (24)	1.81 (0.78, 4.24)	1.80 (0.78, 4.16)	1.79 (0.78, 4.11)

Missing: ever douched (n = 371 non-cases, 1 case), douched in the year prior to enrollment (397 non-cases, 1 case), ever genital talc use (n = 1,033 non-cases, 2 cases), genital talc use in the year prior to enrollment (1,053 non-cases, 2 cases).

^a^Starting at baseline (i.e. enrollment into Sister Study).

^b^Participants with complete confounder information. Pre-baseline cases excluded from the analysis (n = 518).

^c^With age as the primary time scale, starting at age at baseline, with censoring at age at end of follow-up, loss to follow-up or death, whichever occurred first. Adjusted for race/ethnicity (Non-Hispanic White, other), education (yes/no completed college), body mass index (continuous), and age at menarche (continuous).

^d^Additionally adjusted for marital status (ever/never married), age at first pregnancy (nulligravid, < 20, ≥ 20), ever induced abortion, hormonal birth control use (< 2 years, ≥ 2 years), alcohol use (never/former drinker, current drinker), smoking status (ever, never), ever diagnosed with genital warts, another sexually transmitted infection (gonorrhea, herpes, or chlamydia) or pelvic inflammatory disease (yes or no).

^e^Self-reported using douche or genital talc during ages 10–13 or in the year prior to baseline.

In quantitative bias analyses (Supplementary Table 5), we observed that misclassification of cervical cancer would likely bias the observed associations up and away from the null, with substantial loss of precision. For example, when 40% of the cases were re-assigned to be non-cases, the estimated HR for the association between douching in the year prior to enrollment and incident cervical cancer was 2.05 (95% CI 0.64–6.59) rather than HR 2.56 (95% CI 1.10–5.99). The associations between douching at ages 10–13 and pre-baseline cervical cancer were also potentially biased upwards, as were the associations between talc use in the year before baseline and incident cervical cancer, but the HR for talc use at ages 10–13 and pre-baseline cervical cancer was unaffected.

## Discussion

In this large US-based cohort, we observed a positive and statistically significant association between douching and incident cervical cancer, despite a small number of cases. The association between adolescent douching and prior cervical cancer was also positive, though the effect estimate was not statistically significant. We did not see evidence of an association between genital talc use and cervical cancer.

The observed positive association between douching and incident cervical cancer is consistent with previous retrospective case–control studies^23–28^ and prospective studies of the relationship between douching and intermediate outcomes such as HPV infection and cervical lesions^18–22^. Given the known causal role of HPV in cervical cancer development, these results support the hypothesis that douching may alter individuals’ susceptibility or response to the virus. This could occur through the dispersal of foreign pathogens or chemical disruptions to the vaginal microbiome, both of which could alter immune response and thereby facilitate tumor development^11–15^.

We additionally hypothesized that genital talc use could incite an inflammatory response and promote carcinogenesis. However, we saw no evidence of an association between talc use and cervical cancer. As ours is the first study of this topic, additional research, including retrospective case–control comparisons, studies of intermediate outcomes (e.g., HPV infection, cervical lesions), or possibly in vivo or in vitro work, could provide further clarity.

One major limitation of this study is that we are missing data on several potentially important covariates. Specifically, we did not have HPV status, sexual histories, or information about abnormal Pap smears or history of cervical treatments. We adjusted for related variables such as age at first pregnancy, history of induced abortions, marital status, and self-reported history of genital warts or other STIs, but residual confounding may remain. We had no explicit data on use of the HPV vaccine, but our participants (aged 35–74 in 2003–2009) are unlikely to have received it, as Gardasil was approved by the Food and Drug Administration in June 2006 for females aged 9–26^44^.

With 31 incident events, we did not have a sufficient sample size to conduct detailed dose–response analyses or to limit to medically-confirmed cases. Other potential weaknesses include exposure and outcome misclassification, lack of detailed hysterectomy information, and lack of generalizability.

In terms of exposure misclassification, we asked participants about their talc and douching use at ages 10–13 and in the year before study enrollment, and therefore would miss anyone who only used products in the interim. Additionally, women may not be able to accurately remember their use at ages 10–13, which occurred at least 22 years prior to enrollment. Further, because both douching and genital talc use often coincide with menstruation, and earlier menarche may be a risk factor for cervical cancer, this misclassification could be differential with respect to the outcome. More specifically, if women with late menarche (age > 13) were less likely to douche or use talc at ages 10–13 and were less likely to get cervical cancer, our HRs would be attenuated towards the null.

We do not have details on the specific types of products women used. If carcinogenic agents were only present in some products, our effect estimates would be further attenuated. Outcome misclassification is also a concern, especially given our low estimated positive predictive value of 62%. We assessed the potential impact of outcome misclassification using quantitative bias analysis^45^, finding that our reported HRs may be biased up and away from the null. Though this did not change our overall interpretations, the estimates from the bias analysis were less precise.

On the other hand, we observed that some of the women who misreported having cervical cancer actually had precancerous cervical lesions, some of which may have progressed to cancers had they not been treated. This suggests that even if the effects estimated here are not representative of the associations between douching or talc use and cervical cancer, specifically, they may still be capturing information about relationships between health conditions on the same causal pathway. We additionally note that because women with pre-baseline cervical cancer had to survive and be healthy enough to enroll in the Sister Study, women with advanced stage cancers may be under-represented. It is unclear what impact this would have on our estimates.

By including all women who had a hysterectomy, we likely overestimated the number of women at risk for cervical cancer. We showed in sensitivity analyses that not censoring women who had hysterectomies may have attenuated effect estimates. However, as some proportion of these women likely had cervix-sparing subtotal hysterectomies^46,47^, the most accurate effect estimate is likely somewhere in between the two sets of results.

All women in our sample had a sister with breast cancer and therefore the observed associations may not be generalizable to all women or to women in resource poor settings where cervical cancer is a larger public health concern. Though family history of breast cancer is not thought to be related to cervical cancer risk^48^, Sister Study participants are predominantly non-Hispanic White and highly educated, meaning they are likely a low risk group for cervical cancer^49^.

A major strength of the study was our inclusion of both incident and prevalent cervical cancers. The prospective analysis of incident cancer is important to preclude recall bias, which would result if women with cervical cancer were more likely to report exposure. While this type of bias has been observed in at least one retrospective study of genital talc use and ovarian cancer^50,51^, it did not seem to play a role here, as demonstrated by the slightly inverse association we observed between genital talc use and pre-baseline disease. As the possible health consequences of douching are not widely publicized, recall bias seems less likely for that exposure, but it is nevertheless reassuring that the results from the retrospective and prospective analyses are in the same direction.

## Conclusions

In the first prospective study of the relationship between douching and cervical cancer, we observed a positive association between ever or recent douching and incident cervical cancer. These findings were consistent with existing literature on douching and cervical cancer or other related outcomes. In the first epidemiologic study of genital talc use and cervical cancer we did not see evidence of an association, though additional epidemiologic studies may still be warranted.

## Supplementary Information

Supplementary Tables.

## Data Availability

De-identified data is made available upon request as described on the Study Website (https://sisterstudy.niehs.nih.gov/English/data-requests.htm).

## Abbreviations

BMI: Body mass index
CI: Confidence interval
HPV: Human papillomavirus
HR: Hazard ratio
PID: Pelvic inflammatory disease
STI: Sexually transmitted infection
