# Supplementary material for: The association between douching, genital talc use, and the risk of prevalent and incident cervical cancer
Source: Sci Rep. 2021 Jul 21;11:14836. doi: 10.1038/s41598-021-94447-3 (PMC8295379; doi:10.1038/s41598-021-94447-3)
Supplement: Supplementary file 1 — Supplementary Tables. [file 41598_2021_94447_MOESM1_ESM.docx]

**Douching, genital talc use, and prevalent and incident cervical cancer**

Katie M. O’Brien, Clarice R. Weinberg, Aimee A. D’Aloisio, Kristen R. Moore, and Dale P. Sandler

**Supplementary Table 1. Characteristics of women who used genital talc or douched during ages 10-13 (n=49,353^a^)**

|  | **Non-users**  **(n= 39,229; 79%)** | **Douched^b^**  **(n= 1,555; 3%)** | **Used genital talc^b^**  **(n= 9,269; 19%)** |
| --- | --- | --- | --- |
| **Age at baseline; mean (sd)** | 55.8 (9.1) | 52.7 (8.0) | 55.8 (8.4) |
| **Baseline BMI (kg/m^2^); mean (sd)** | 27.5 (6.1) | 29.5 (7.1) | 28.8 (6.6) |
| **BMI ages 30-39 (kg/m^2^); mean (sd)** | 23.1 (3.8) | 24.5 (5.3) | 23.8 (4.3) |
| **Age at menarche; mean (sd)** | 12.7 (1.5) | 12.3 (1.6) | 12.3 (1.5) |
| **Race; N (%)** |  |  |  |
| Non-Hispanic White | 33,629 (86) | 1,067 (69) | 7,286 (79) |
| Non-Hispanic Black/African-American | 2,545 (6) | 385 (25) | 1,432 (15) |
| Hispanic/Latina | 1,982 (5) | 63 (4) | 353 (4) |
| Other | 1,065 (3) | 39 (3) | 195 (2) |
| **Childhood SES^c^; N (%)** |  |  |  |
| Well off | 2,601 (7) | 73 (5) | 486 (5) |
| Middle income | 23,450 (60) | 914 (59) | 5,420 (59) |
| Low income | 10,028 (26) | 424 (27) | 2557 (28) |
| Poor | 3,061 (8) | 142 (9) | 782 (8) |
| **Education; N (%)** |  |  |  |
| High school or less | 6,062 (15) | 286 (18) | 1,376 (15) |
| Some college | 13,026 (33) | 621 (40) | 3,278 (35) |
| College graduate | 10,692 (27) | 350 (22) | 2,415 (26) |
| Graduate degree | 9,442 (24) | 298 (19) | 2,198 (24) |
| **Marital status; N (%)** |  |  |  |
| Never married | 2,045 (5) | 90 (6) | 550 (6) |
| Married/living as married | 29,541 (75) | 1,098 (71) | 6,802 (73) |
| Divorced/widowed/separated | 7,636 (19) | 366 (24) | 1,913 (21) |
| **Pap smear in year prior to enrollment; N (%)** | 29,925 (76) | 1,210 (78) | 7,126 (77) |
| **Age at menarche; N (%)** |  |  |  |
| <12 | 7,262 (19) | 455 (29) | 2,598 (28) |
| 12-13 | 21,979 (56) | 822 (53) | 5,260 (57) |
| >14 | 9,950 (25) | 277 (18) | 1,406 (15) |
| **Parity; N (%)** |  |  |  |
| Nulliparous | 7,056 (18) | 298 (19) | 1,712 (18) |
| 1 child | 5,568 (14) | 308 (20) | 1,358 (15) |
| 2 children | 14,376 (37) | 558 (36) | 3,478 (38) |
| >2 children | 12,211 (31) | 386 (25) | 2,710 (29) |
| **Age at first pregnancy; N (%)** |  |  |  |
| Nulligravid | 4,908 (13) | 179 (12) | 1,180 (13) |
| <20 | 7,104 (18) | 461 (30) | 1,965 (21) |
| 20-24 | 13,660 (35) | 500 (32) | 3,259 (35) |
| 25-29 | 8,625 (22) | 242 (16) | 1,853 (20) |
| >30 | 4,865 (12) | 164 (11) | 992 (11) |
| **Induced abortion; N (%)** | 5,883 (15) | 371 (24) | 1,537 (17) |
| **Duration of hormonal birth control; N (%)** |  |  |  |
| Never user | 6,035 (15) | 186 (12) | 1,312 (14) |
| 0-<2 years | 5,988 (15) | 224 (14) | 1,388 (15) |
| 2-<10 years | 16,699 (43) | 630 (41) | 4,088 (44) |
| >10 years | 10,471 (27) | 511 (33) | 2,471 (27) |
| **Alcohol use; N (%)** |  |  |  |
| Never or former drinker | 7,360 (19) | 349 (22) | 1,832 (20) |
| Current drinker, <1 drink/day | 26,393 (67) | 1,003 (65) | 6,331 (68) |
| Current drinker, >1 drink/day | 5,462 (14) | 202 (13) | 1,101 (12) |
| **Started drinking regularly <age 13** | 1,535 (4) | 124 (8) | 417 (5) |
| **Smoking; N (%)** |  |  |  |
| Never | 22,195 (57) | 744 (48) | 5,069 (55) |
| Former | 13,914 (35) | 620 (40) | 3,411 (37) |
| Current | 3,112 (8) | 191 (12) | 784 (8) |
| **Started smoking <age 13** | 1,091 (3) | 114 (7) | 336 (4) |
| **Weight relative to peers, age 10; N (%)** |  |  |  |
| Lighter | 14,044 (36) | 497 (32) | 2,968 (32) |
| Same weight | 18,518 (47) | 702 (45) | 4,159 (45) |
| Heavier | 6,641 (17) | 355 (23) | 2,137 (23) |
| **Ever had genital warts; N (%)** | 2,311 (6) | 137 (9) | 554 (6) |
| Age at diagnosis; mean (sd) | 28.8 (9.5) | 28.5 (10.5) | 29.4 (10.0) |
| **Ever had an STI^d^; N (%)** | 4,451 (11) | 286 (18) | 1,129 (12) |
| Age at diagnosis; mean (sd) | 29.5 (10.0) | 26.1 (8.7) | 28.8 (9.6) |
| **Ever had PID; N (%)** | 1,310 (3) | 91 (6) | 419 (5) |
| Age at diagnosis; mean (sd) | 28.0 (8.0) | 25.9 (8.6) | 27.5 (8.1) |
| **Postmenopausal; N (%)** | 25,886 (66) | 907 (58) | 6,492 (70) |
| **Hysterectomy; N (%)** | 11,900 (30) | 583 (37) | 3,370 (36) |
| **Oophorectomy; N (%)** | 6,877 (18) | 315 (20) | 1,900 (21) |
| **Exposed to diethylstilbestrol *in utero*^e^; N (%)** | 906 (2) | 38 (2) | 219 (2) |
| **Family history of cervical cancer^f^; N (%)** | 1,490 (4) | 66 (4) | 372 (4) |

Abbreviations: BMI=body mass index; SES=socioeconomic status; PID=pelvic inflammatory disease

Missing values: BMI (10 non-users, 1 doucher, 4 talc users), BMI ages 30-39 (343 non-users, 12 douchers, 49 talc users)age at menarche (38 non-users, 1 doucher, 5 talc users), Race/ethnicity (6 non-users, 1 doucher, 3 talc users), childhood socioeconomic status (89 non-users, 2 douchers), education (7 non-users, 2 talc users), marital status (7 non-users, 1 doucher, 4 talc users), Pap smear in year prior to enrollment (34 non-users, 5 talc users), parity (18 non-users, 5 douchers, 11 talc users), age at first pregnancy (67 non-users, 9 douchers, 20 talc users), induced abortion (18 non-users, 5 douchers, 11 talc users), duration of hormonal contraceptive use (36 non-users, 4 douchers, 10 talc users), alcohol use (14 non-users, 1 doucher, 5 talc users), started drinking alcohol regularly, age 13 (10 non-users, 2 douchers, 5 talc users), smoking status (8 non-users, 5 talc users), started smoking < age 13 (9 non-users, 2 douchers, 7 talc users), weight relative to peers (26 non-users, 1 doucher, 5 talc users), ever had genital warts (32 non-users, 4 talc users), ever had an STI (92 non-users, 7 douchers, 25 talc users), ever had PID (63 non-users, 2 douchers, 13 talc users), hysterectomy (22 non-users, 1 talc user), oophorectomy (64 non-users, 5 douchers, 11 talc users)

^a^Excluded if: withdrawn from study (n=3), cervical cancer status unknown (n=3), uncertain age at diagnosis (n=4), diagnosed before age 13 (n=1), did not fill out questionnaire on use of talc or douching products (n=1,365), were missing data for both genital talc use and douching at ages 10-13 (n=155)

^b^Non-exclusive categories

^c^Defined as family’s income level during the majority of time growing up

^d^STI= sexually transmitted infection, here defined as self-reported gonorrhea, herpes, chlamydia

^e^If responded that mother “definitely” or “probably” took diethylstilbestrol; all others assumed to be unexposed

^f^Had at least one first degree relative (sister, mother, daughter)

**Supplementary Table 2. Characteristics of women who used genital talc or douched in the year prior to baseline (total n=49,500)**

|  | **Non-users**  **(n= 37,156; 75%)** | **Douched^b^**  **(n= 6,712; 14%)** | **Used genital talc^b^**  **(n= 7,186; 15%)** |
| --- | --- | --- | --- |
| **Age at baseline; mean (sd)** | 56.0 (9.0) | 54.0 (8.8) | 55.5 (9.0) |
| **Baseline BMI (kg/m^2^); mean (sd)** | 27.3 (6.0) | 29.2 (6.7) | 29.8 (6.9) |
| **BMI ages 30-39 (kg/m^2^); mean (sd)** | 23.0 (3.7) | 24.0 (4.8) | 24.2 (4.6) |
| **Age at menarche; mean (sd)** | 12.7 (1.5) | 12.6 (1.6) | 12.5 (1.5) |
| **Race; N (%)** |  |  |  |
| Non-Hispanic White | 32,285 (87) | 4,470 (67) | 5,871 (82) |
| Non-Hispanic Black/African-American | 2,240 (6) | 1,530 (23) | 805 (11) |
| Hispanic/Latina | 1,657 (4) | 509 (8) | 339 (5) |
| Other | 965 (3) | 202 (3) | 168 (2) |
| **Childhood SES^c^; N (%)** |  |  |  |
| Well off | 2,497 (7) | 308 (5) | 400 (6) |
| Middle income | 22,369 (60) | 3,737 (56) | 4,263 (59) |
| Low income | 9,426 (25) | 1,926 (29) | 1,910 (27) |
| Poor | 2,774 (7) | 725 (11) | 598 (8) |
| **Education; N (%)** |  |  |  |
| High school or less | 5,323 (14) | 1,427 (21) | 1,234 (17) |
| Some college | 11,832 (32) | 2,797 (42) | 2,751 (38) |
| College graduate | 10,470 (28) | 1,420 (21) | 1,752 (24) |
| Graduate degree | 9,524 (26) | 1,068 (16) | 1,447 (20) |
| **Marital status; N (%)** |  |  |  |
| Never married | 1,909 (5) | 439 (7) | 407 (6) |
| Married/living as married | 28,270 (76) | 4,590 (68) | 5,271 (73) |
| Divorced/widowed/separated | 6,970 (19) | 1,681 (25) | 1,506 (21) |
| **Pap smear in year prior to enrollment; N (%)** | 28,447 (77) | 5,179 (77) | 5,412 (75) |
| **Age at menarche; N (%)** |  |  |  |
| <12 | 7,353 (20) | 1,479 (22) | 1,636 (23) |
| 12-13 | 21,061 (57) | 3,550 (53) | 3,992 (56) |
| >14 | 8,721 (23) | 1,670 (25) | 1,546 (22) |
| **Parity; N (%)** |  |  |  |
| Nulliparous | 6,723 (18) | 1,250 (19) | 1,265 (18) |
| 1 child | 5,229 (14) | 1,166 (17) | 1,014 (14) |
| 2 children | 13,759 (37) | 2,371 (35) | 2,611 (36) |
| >2 children | 11,430 (31) | 1,913 (29) | 2,289 (32) |
| **Age at first pregnancy; N (%)** |  |  |  |
| Nulligravid | 4,695 (13) | 804 (12) | 879 (12) |
| <20 | 6,281 (17) | 1,971 (29) | 1,588 (22) |
| 20-24 | 12,908 (35) | 2,246 (34) | 2,586 (36) |
| 25-29 | 8,379 (23) | 1,120 (17) | 1,412 (20) |
| >30 | 4,837 (13) | 546 (8) | 708 (10) |
| **Induced abortion; N (%)** | 5,602 (15) | 1,189 (18) | 1,142 (16) |
| **Duration of hormonal birth control; N (%)** |  |  |  |
| Never user | 5,708 (15) | 935 (14) | 1,076 (15) |
| 0-<2 years | 5,683 (15) | 959 (14) | 1,093 (15) |
| 2-<10 years | 15,982 (43) | 2,815 (42) | 3,029 (42) |
| >10 years | 9,745 (26) | 1,998 (30) | 1,981 (28) |
| **Alcohol use; N (%)** |  |  |  |
| Never or former drinker | 6,822 (18) | 1,475 (22) | 1,487 (21) |
| Current drinker, <1 drink/day | 25,032 (67) | 4,490 (67) | 4,864 (68) |
| Current drinker, >1 drink/day | 5,289 (14) | 746 (11) | 829 (12) |
| **Started drinking regularly <age 13** | 1,486 (4) | 310 (5) | 286 (4) |
| **Smoking; N (%)** |  |  |  |
| Never | 21,059 (57) | 3,513 (52) | 3,997 (56) |
| Former | 13,348 (36) | 2,331 (35) | 2,571 (36) |
| Current | 2,839 (7) | 867 (13) | 615 (9) |
| **Started smoking <age 13** | 1,046 (3) | 284 (4) | 226 (3) |
| **Weight relative to peers, age 10; N (%)** |  |  |  |
| Lighter | 13,137 (35) | 2,446 (36) | 2,293 (32) |
| Same weight | 17,498 (47) | 3,078 (46) | 3,261 (45) |
| Heavier | 6,504 (18) | 1,180 (18) | 1,624 (23) |
| **Ever had genital warts; N (%)** | 2,240 (6) | 385 (6) | 418 (6) |
| Age at diagnosis; mean (sd) | 29.0 (9.7) | 27.6 (8.9) | 29.4 (9.8) |
| **Ever had an STI^d^; N (%)** | 4,202 (11) | 918 (14) | 831 (12) |
| Age at diagnosis; mean (sd) | 29.3 (10.0) | 28.7 (9.6) | 29.4 (9.9) |
| **Ever had PID; N (%)** | 1,240 (3) | 335 (5) | 292 (4) |
| Age at diagnosis; mean (sd) | 27.9 (8.1) | 27.3 (7.5) | 28.3 (8.4) |
| **Postmenopausal; N (%)** | 25,227 (68) | 3,908 (58) | 4,686 (65) |
| **Hysterectomy; N (%)** | 11,221 (30) | 2,466 (37) | 2,518 (35) |
| **Oophorectomy; N (%)** | 6,523 (18) | 1,310 (20) | 1,442 (20) |
| **Exposed to diethylstilbestrol *in utero*^e^; N (%)** | 872 (2) | 147 (2) | 172 (2) |
| **Family history of cervical cancer^f^; N (%)** | 1,383 (4) | 310 (5) | 278 (4) |

Abbreviations: BMI=body mass index; SES=socioeconomic status; PID=pelvic inflammatory disease

Missing values: BMI (13 non-users, 2 talc users), BMI ages 30-39 (291 non-users, 72 douchers, 61 talc users) age at menarche (21 non-users, 13 douchers, 12 talc users), Race/ethnicity (9 non-users, 1 doucher, 3 talc users), childhood socioeconomic status (90 non-users, 16 douchers, 15 talc users), education (7 non-users, 2 talc users), marital status (7 non-users. 2 douchers, 2 talc users), Pap smear in year prior to enrollment (29 non-users, 7 douchers, 5 talc users), parity (15 non-users, 12 douchers, 7 talc users), age at first pregnancy (56 non-users, 25 douchers, 13 talc users), induced abortion (15 non-users, 12 douchers, 7 talc users), duration of hormonal contraceptive use (38 non-users, 5 douchers, 7 talc users), alcohol use (13 non-users, 1 doucher, 6 talc users), started drinking alcohol regularly, age 13 (12 non-users, 1 doucher, 4 talc users), smoking status (10 non-users, 1 doucher, 3 talc users), started smoking <age 13 (12 non-users, 2 douche users, 4 talc users), weight relative to peers (17 non-users, 8 douchers, 8 talc users), ever had genital warts (28 non-users, 4 douchers, 4 talc users), ever had an STI (81 non-users, 24 douchers, 18 talc users), ever had PID (57 non-users, 11 douchers, 10 talc users), hysterectomy (17 non-users, 3 douchers, 4 talc users), oophorectomy (49 non-users, 13 douchers, 19 talc users)

^a^Excluded if: withdrawn from study (n=3), cervical cancer status unknown (n=3), uncertain age at diagnosis (n=4), diagnosed before age 13 (n=1), did not fill out questionnaire on use of talc or douching products (n=1,365), were missing data for both genital talc use and douching in the year prior to enrollment (n=8)

^b^Non-exclusive categories

^c^Defined as family’s income level during the majority of time growing up

^d^STI=sexually transmitted infection, here defined as self-reported gonorrhea, herpes, chlamydia

^e^If responded that mother “definitely” or “probably” took diethylstilbestrol; all others assumed to be unexposed

^f^Had at least one first degree relative (sister, mother, daughter)

**Supplementary Table 3.** **Associations between douching and genital talc use during ages 10-13 and pre-baseline cervical cancer with censoring at age at hysterectomy (n=49,302^a^)**

|  | **Person-time at risk** (years)^a^ | **Non-cases; N(%)**  N=48,814^b^ | **Cervical cancer cases, N(%)**  N=488^b^ | | **Age-adjusted**  **Hazard Ratio (95% CI)** | **Adjusted Hazard Ratio**^c^  **(95% CI)** | **Fully adjusted Hazard Ratio**^c,d^  **(95% CI)** |
| --- | --- | --- | --- | --- | --- | --- | --- |
| Douching, ages 10-13 | | | | | | |  |
| Ever | | | | | | |  |
| No | 1,758,283 | 46,586 (97) | 462 (95) | 1.00 | | 1.00 | 1.00 |
| Yes | 52,176 | 1,525 (3) | 22 (5) | 1.58 (1.03, 2.43) | | 1.64 (1.07, 2.52) | 1.49 (0.97, 2.29) |
| Frequency |  |  |  |  | |  |  |
| None | 1,758,283 | 46,586 (97) | 462 (95) | 1.00 | | 1.00 | 1.00 |
| Sometimes | 47,364 | 1,378 (3) | 19 (4) | 1.50 (0.95, 2.38) | | 1.55 (0.98, 2.46) | 1.42 (0.89, 2.25) |
| Frequently | 4,812 | 147 (0) | 3 (1) | 2.38 (0.76, 7.43) | | 2.57 (0.82, 8.07) | 2.19 (0.70, 6.86) |
|  |  |  |  | p-trend=0.02 | | p-trend=0.02 | p-trend=0.05 |
| Genital talc use, ages 10-13 | | | | | | |  |
| Ever | | | | | | |  |
| No | 1,393,878 | 36,931 (80) | 373 (81) | 1.00 | | 1.00 | 1.00 |
| Yes | 337,880 | 9,138 (20) | 89 (19) | 0.98 (0.78, 1.24) | | 0.98 (0.77, 1.24) | 0.96 (0.76, 1.21) |
| Frequency |  |  |  |  | |  |  |
| None | 1,393,878 | 36,931 (80) | 373 (81) | 1.00 | | 1.00 | 1.00 |
| Sometimes | 287,852 | 7,758 (17) | 74 (16) | 0.96 (0.75, 1.23) | | 0.95 (0.74, 1.22) | 0.93 (0.73, 1.20) |
| Frequently | 50,029 | 1,380 (3) | 15 (3) | 1.13 (0.67, 1.89) | | 1.12 (0.66, 1.89) | 1.11 (0.65, 1.87) |
|  |  |  |  | p-trend=0.96 | | p-trend=1.00 | p-trend=0.89 |
| Combined ever use, ages 10-13 | | | | | | |  |
| Neither | 1,361,924 | 36,032 (79) | 363 (79) | 1.00 | | 1.00 | 1.00 |
| Both | 23,651 | 688 (2) | 8 (2) | 1.25 (0.62, 2.52) | | 1.30 (0.65, 2.62) | 1.16 (0.58, 2.34) |
| Talc/no douching | 306,670 | 8,245 (18) | 80 (18) | 0.98 (0.77, 1.25) | | 0.97 (0.76, 1.24) | 0.96 (0.75, 1.22) |
| Douching/no talc | 18,992 | 552 (1) | 9 (2) | 1.76 (0.91, 3.41) | | 1.82 (0.93, 3.54) | 1.56 (0.80, 3.05) |

Missing: douching (n=703 non-cases, 4 cases), genital talc use (n=2,745 non-cases, 26 cases), both (n=3,297 non-cases, 28 cases)

^a^Starting at age 13, censoring at minimum of age at baseline or age at hysterectomy

^b^Participants with complete confounder information

^c^With age as the primary time scale, adjusting for weight relative to peers at age 10 (ordinal), race/ethnicity (non-Hispanic White, non-Hispanic Black/African-American, Latina/Hispanic, or other), childhood socioeconomic status (well off, middle income, low income, poor), and age at menarche (<12, 12-13 >14) *in utero* diethylstilbestrol exposure (yes or no).

^d^Additionally adjusted for *in utero* diethylstilbestrol exposure (yes or no), regular drinking before age 14 (yes/no), and smoking before age 14 (yes/no).

**Supplementary Table 4.** **Associations between ever douched and genital talc use and incident cervical cancer, excluding those with hysterectomy prior to baseline and censoring at age of hysterectomy** **(n=33,395)**

|  | | **Person-time (years)^a^** | | **Non-cases; N(%)**  N=33,396^b^ | **Incident cervical cancer cases, N(%)**  N=29^b^ | **Age-adjusted**  **Hazard Ratio (95% CI)** | **Adjusted Hazard Ratio**^c^ **(95% CI)** | **Fully-adjusted Hazard Ratio**^c,d^ **(95% CI)** |
| --- | --- | --- | --- | --- | --- | --- | --- | --- |
| Ever douched^e^ | | | | | | |  |  |
| No | | 261,715 | | 28,259 (85) | 18 (64) | 1.00 | 1.00 | 1.00 |
| Yes | | 43,780 | | 4,851 (15) | 10 (36) | 3.34 (1.54, 7.27) | 3.22 (1.36, 7.63) | 2.89 (1.20, 6.94) |
| Douched in the year prior to enrollment | | | | | | |  |  |
| No | 268,336 | | 28,933 (87) | | 19 (68) | 1.00 | 1.00 | 1.00 |
| Yes | 37,043 | | 4,163 (13) | | 9 (32) | 3.45 (1.57, 7.60) | 3.28 (1.37, 7.87) | 2.97 (1.23, 7.16) |
| Ever genital talc use^e^ | | | | | | |  |  |
| No | | 220,817 | | 24,066 (74) | 19 (70) | 1.00 | 1.00 | 1.00 |
| Yes | | 79,781 | | 8,631 (26) | 8 (30) | 1.16 (0.51, 2.67) | 1.18 (0.54, 2.61) | 1.17 (0.53, 2.56) |
| Genital talc use in the year prior to enrollment | | | | | |  |  |  |
| No | | 258,183 | | 28,094 (86) | 21 (78) | 1.00 | 1.00 | 1.00 |
| Yes | | 42,307 | | 4,592 (14) | 6 (22) | 1.74 (0.70, 4.29) | 1.71 (0.70, 4.16) | 1.68 (0.69, 4.05) |

Missing: ever douched (257 non-cases, 1 case), douched in the year prior to enrollment (271 non-cases, 1 case), ever genital talc use (670 non-cases, 2 cases), genital talc use in the year prior to enrollment (681 non-cases, 2 cases)

^a^Starting at baseline (i.e. enrollment into Sister Study)

^b^Participants with complete confounder information. Pre-baseline cases excluded from the analysis (n=518).

^c^With age as the primary time scale, starting at age at baseline, with censoring at age of hysterectomy, end of follow-up, loss to follow-up, or death, whichever occurred first. Adjusted for race/ethnicity (non-Hispanic White, other), education (yes/no completed college), body mass index (continuous), menopausal status (pre or postmenopausal), age at menarche (continuous), and first-degree family history of cervical cancer (yes or no).

^d^Additionally adjusted for marital status (ever/never married), age at first pregnancy (nulligravid, <20, >20), ever induced abortion, duration of hormonal birth control use (none, <2 years, >2 years), alcohol use (never/former drinker, current drinker), smoking status (ever, never), ever diagnosed with genital warts (yes or no), and ever diagnosed with another sexually transmitted infection (gonorrhea, herpes, or chlamydia) or pelvic inflammatory disease (yes or no)

^e^Self-reported using douche or genital talc during ages 10-13 or in the year prior to enrollment

**Supplementary Table 5. Bias analysis**

| **Exposure measurement** | **Outcome measurement** | **% of cases randomly re-assigned to be non-cases** | **Original fully-adjusted estimate** | **Revised fully-adjusted estimate** |
| --- | --- | --- | --- | --- |
| Douching,  ages 10-13 | Pre-baseline cervical cancer | 40% | 1.32 (0.86, 2.03) | 1.26 (0.63, 2.51) |
| Douching,  ages 10-13 | Pre-baseline cervical cancer | 20% | 1.32 (0.86, 2.03) | 1.34 (0.80, 2.24) |
| Genital talc use,  ages 10-13 | Pre-baseline cervical cancer | 40% | 0.95 (0.76, 1.19) | 0.96 (0.66, 1.39) |
| Genital talc use,  ages 10-13 | Pre-baseline cervical cancer | 20% | 0.95 (0.76, 1.19) | 0.94 (0.71, 1.24) |
| Douching in the year prior to enrollment | Incident cervical cancer | 40% | 2.56 (1.10, 5.99) | 2.05 (0.64, 6.59) |
| Douching in the year prior to enrollment | Incident cervical cancer | 20% | 2.56 (1.10, 5.99) | 2.32 (0.90, 5.96) |
| Genital talc use in the year prior to enrollment | Incident cervical cancer | 40% | 1.79 (0.78, 4.11) | 1.52 (0.55, 4.24) |
| Genital talc use in the year prior to enrollment | Incident cervical cancer | 20% | 1.79 (0.78, 4.11) | 1.65 (0.63, 4.32) |
